# Lactate Metabolism and Signaling in Amyotrophic Lateral Sclerosis

**DOI:** 10.3390/cells15171571

**Published:** 2026-08-29

**Authors:** Xiaonan Ma, Jinmeng Liu, Yingjun Guan, Chunjie Xu, Mu Li, Xue Zhang, Zongze Li, Lanli Zhang, Xuemei Wang, Haoyun Zhang, Yanchun Chen

**Affiliations:** 1 Department of Histology and Embryology, Shandong Second Medical University, Weifang 261053, China; 18855241172@163.com (X.M.); xu1261781793@163.com (C.X.); zhangxue12171029@163.com (X.Z.); 15321501229@163.com (Z.L.); m19506525100@163.com (L.Z.); 18863662168@163.com (X.W.); haoyunzh@sdsmu.edu.cn (H.Z.); 2 Neurologic Disorders and Regenerative Repair Lab of Shandong Higher Education, Shandong Second Medical University, Weifang 261053, China; liujinmeng@sdsmu.edu.cn (J.L.); limu1009@gmail.com (M.L.); 3 Department of Physiology and Pathophysiology, School of Basic Medicine, Qingdao University, Qingdao 266000, China

**Keywords:** amyotrophic lateral sclerosis, lactate metabolism, lactate signaling, lactylation, therapy

## Abstract

Amyotrophic lateral sclerosis (ALS) is a progressive and fatal neurodegenerative disease characterized by the selective degeneration of upper and lower motor neurons (MNs). Although the specific pathogenesis of ALS is not yet fully understood, there is increasing evidence that abnormal energy metabolism plays a key role in the onset and progression of the disease. Lactate has traditionally been considered a metabolic by-product of glycolysis and has received increasing attention in recent years. Research has shown that lactate is not only an important energy substrate but also a signaling molecule that regulates a variety of physiological processes, including neuron-glia metabolic coupling, neuroprotection and inflammatory responses. In ALS, abnormalities in lactate metabolism, dysfunction of the lactate shuttle, and dysregulation of lactate-related signaling pathways may jointly lead to neuronal energy deficits and increased neuroinflammation, thereby promoting MN degeneration. This review summarizes the latest advances in lactate metabolism and lactate-mediated signaling in ALS, with particular emphasis on their roles in neuronal energy regulation, neuroprotection and inflammatory regulation. In addition, we also discuss potential therapeutic strategies for lactate metabolism and its related pathways, aiming to provide new insights into the pathogenesis of ALS and the development of treatment methods for lactate-related metabolism.

## 1. Introduction

Amyotrophic lateral sclerosis (ALS) is a fatal and progressive neurodegenerative disease that selectively attacks upper and lower motor neurons (MNs) and leads to loss of function [1]. The pathological process mainly affects the spinal cord, brainstem and motor cortex, clinically leading to muscle weakness, muscular atrophy, and ultimately paralysis. Most patients eventually die of respiratory failure within 3 to 5 years after the onset of symptoms [2]. Although extensive research has been conducted, unfortunately, the etiology of ALS remains largely unknown. Current evidence suggests that the pathogenesis of this disease is multifactorial and highly complex. Studies have found a variety of pathological mechanisms in ALS, including the accumulation of neurotoxic substances, protein misfolding and aggregation, impaired axonal transport, mitochondrial dysfunction, oxidative stress and neuroinflammation, and insufficient neurotrophic support [3,4,5,6]. However, the precise molecular mechanisms underlying MN degeneration have not been fully elucidated. At present, available treatment strategies for ALS are mainly symptomatic, which can only moderately delay the progression of the disease and improve patients’ quality of life to a certain extent. However, effective disease-modifying therapies are still lacking [7]. Of note, systemic metabolic abnormalities, including progressive weight loss and energy homeostasis imbalance, are commonly observed in ALS patients. Importantly, these metabolic perturbations are not merely secondary consequences of motor disability; rather, they represent integral components of ALS pathophysiology, involving complex crosstalk between the central nervous system (CNS) and peripheral metabolic tissues [8]. Increasing evidence suggests that dysregulation of cellular energy metabolism is one of the crucial mechanisms in the pathogenesis of ALS. Metabolic abnormalities are not limited to MNs but also involve surrounding glial cells, including astrocytes and oligodendrocytes, as well as peripheral tissues such as skeletal muscle and liver, eventually leading to systemic metabolic imbalance [6,9,10,11]. This mechanistic perspective has spurred growing interest in metabolic and nutritional interventions. Indeed, accumulating clinical evidence indicates that nutritional support strategies, particularly high-calorie supplementation, can significantly improve patient outcomes and prolong survival. Of note, post hoc analyses from clinical trials have suggested that these benefits may be more pronounced in rapidly progressive cases [12].

MNs are characterized by large cell bodies, long axons, and sustained electrical activity, which bring extremely high energy requirements [8]. In the CNS, neurons primarily rely on glucose transporter 3 (GLUT3) for glucose uptake [13]. However, there is evidence that glucose alone may not be sufficient to support the large energy needs of MNs [14]. MNs predominantly express monocarboxylate transporter 2 (MCT2) and promote efficient uptake of lactate provided by surrounding glial cells, including astrocytes and oligodendrocytes [15]. In other words, neurons can use lactate from astrocytes as an important energy substrate, especially at the peak of neuronal activity [16].

Lactic acid was first isolated from yogurt by *Carl Wilhelm Scheele* in 1780 and exists in two stereoisomeric forms, L-lactate and D-lactate. At the physiological pH of approximately 7.4, lactic acid is almost completely dissociated to form lactate [17]. Although lactate is a normal metabolite in both resting and exercising tissues, its excessive accumulation in the blood has important clinical significance [18]. Elevated lactate levels, or hyperlactatemia, are usually associated with conditions such as sepsis, hypoxia, shock, and mitochondrial dysfunction [19,20]. Elevated blood lactate levels may lead to metabolic acidosis. As an important biomarker for detecting tissue hypoperfusion and hypoxia, this is the most important clinical application of lactate.

Although lactate is widely used as an important clinical indicator for assessing disease severity, it has long been considered a metabolic “waste product” generated during glycolysis. However, increasing evidence in recent years has challenged this traditional view and revealed that lactate plays an important role in cellular metabolism [18]. One important function of lactate production is to regenerate NAD^+^ through the reduction of pyruvate by lactate dehydrogenase (LDH), thereby uncoupling glycolysis from mitochondrial oxidative metabolism. This process allows glycolysis to proceed independently of the tricarboxylic acid (TCA) cycle, enabling continued ATP production even when mitochondrial oxidative capacity is limited [21]. If lactate production is insufficient, the regeneration of NAD^+^ is compromised, which can limit glycolytic ATP production under conditions of high energy demand. The presence of monocarboxylate transporters (MCTs) enables lactate transport between cells, thereby promoting metabolic coupling between cells and tissues [22]. In addition to its metabolic role, lactate is now recognized as an important signaling molecule involved in intracellular and intercellular communication and has been shown to be involved in protein post-translational modification processes such as lactylation [23,24,25].

According to the astrocyte-neuron lactate shuttle (ANLS) hypothesis, astrocytes take up glucose and undergo aerobic glycolysis to produce lactate, which is subsequently released into the extracellular space through MCTs [16,26,27,28,29]. Neurons then import lactate through membrane-bound MCTs, and lactate is converted into pyruvate by LDH, which enters the mitochondrial TCA cycle to generate ATP. This metabolic cooperation enables astrocytes to rapidly provide energy substrates, while neurons preferentially utilize oxidative metabolism to efficiently meet their high energy needs during intense synaptic activity [28,30]. Recent studies show that exogenous lactate supplementation or metabolic pathway modulation exerts neuroprotective effects in ALS cellular and animal models [31,32]. Notably, lactate metabolic dysregulation has been observed in multiple neurodegenerative diseases, including Alzheimer’s disease (AD) [33], Parkinson’s disease (PD) [34], and ALS. Although the manifestation patterns of lactate metabolic abnormalities may differ across these diseases, their role as a shared metabolic feature points to potential therapeutic targets [35]. Therefore, understanding the roles of lactate metabolism and neuron–glia metabolic coupling in ALS may provide new insights into disease mechanisms and potential metabolic therapy strategies.

## 2. Lactate Metabolism and Physiological Functions

### 2.1. Lactate Production

**Figure 1:**
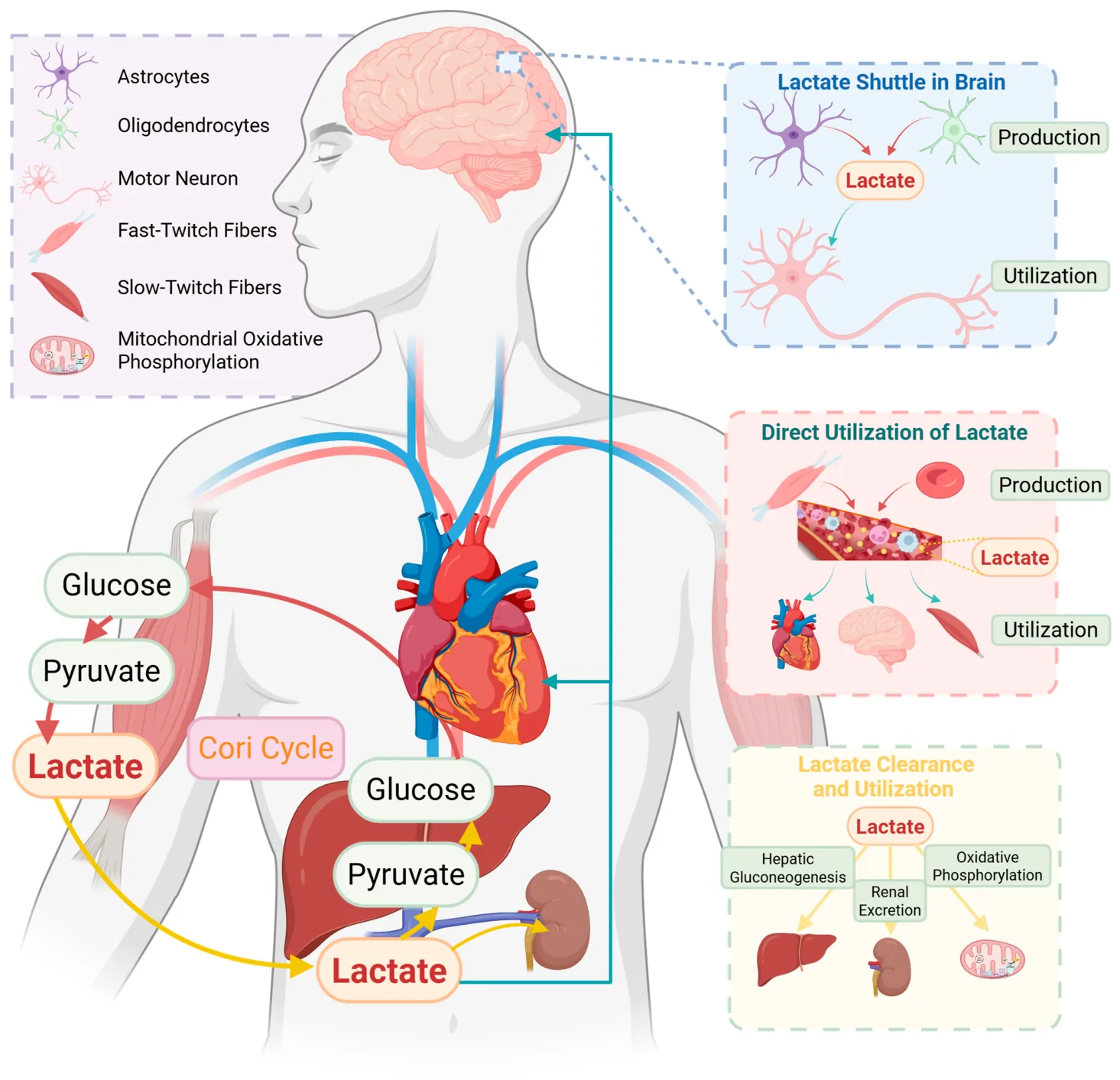
Schematic diagram of lactate metabolism, shuttling, and excretion. The lactate shuttle system in the brain primarily involves two pathways: the astrocyte-motor neuron axis and the oligodendrocyte-neuron axis. Lactate of peripheral origin is mainly produced by fast-twitch muscle fibers and red blood cells, and its clearance depends on three pathways: the Cori cycle (gluconeogenesis) in the liver, renal excretion, and intracellular oxidative phosphorylation. At the same time, tissues such as cardiomyocytes and neurons can directly uptake and utilize lactate as an energy substrate.

A variety of tissues in the human body produce lactate, including skeletal muscle during strenuous exercise, intestinal tissue, skin, brain astrocytes, red blood cells, and various tumor tissues [36]. In the CNS, astrocytes and oligodendrocytes generate lactate through aerobic glycolysis, which is then transported to neurons and used as an energy substrate for ATP production [37] (Figure 1). High levels of glycolytic activity in skeletal muscle during strenuous exercise can lead to rapid accumulation of lactate and cause a temporary increase in circulating lactate levels [38]. In pathological conditions such as myocardial infarction or shock, tissue hypoxia can induce metabolism to turn to anaerobic glycolysis, resulting in increased lactate production [39]. It is worth noting that mature red blood cells are unique in that they lack mitochondria; therefore, their energy metabolism is completely dependent on glycolysis, and lactate is continuously produced even under normoxic conditions. In addition, many tumor cells exhibit a high metabolic state, and they still exhibit high levels of lactate production when oxygen is sufficient, a metabolic phenomenon known as the Warburg effect [40].

### 2.2. Lactate Clearance and Utilization

Lactate production and clearance in the human body are maintained in a dynamic balance. The main pathways of lactate clearance include hepatic gluconeogenesis, renal metabolic excretion and direct oxidative utilization of various tissues.

The liver is an important site of lactate clearance, primarily via the gluconeogenic pathway of the Cori cycle [41,42] (Figure 1). In this pathway, LDH oxidizes lactate back to pyruvate, and then pyruvate regenerates glucose through a series of enzymatic reactions. The newly synthesized glucose is released into the bloodstream for use by peripheral tissues such as the brain and skeletal muscle. The conversion of lactate to glucose is particularly important during exercise, fasting and major diseases because of the high glucose demand and elevated lactate levels under such conditions [41]. The Cori cycle is a key metabolic pathway linking anaerobic metabolism of skeletal muscle with hepatic gluconeogenesis. Despite the existence of net energy consumption, this expenditure is necessary and worthwhile for rapid access to energy in emergencies. Therefore, hepatic gluconeogenesis is an important way to remove lactate, which not only contributes to lactate clearance but also promotes systemic glucose homeostasis.

The kidney also serves as an important site of lactate clearance, with its contribution becoming particularly pronounced during strenuous exercise or hyperlactatemia. Under conditions of marked hyperlactatemia, the kidneys begin to excrete lactate directly into the urine. However, this direct excretion accounts for only a minor fraction (approximately 10–12%) of total renal lactate clearance, with the majority being removed through metabolic utilization such as gluconeogenesis [43]. More importantly, the renal cortex has a significant gluconeogenic capacity to convert lactate into glucose, like the liver, which is one of the main sources of blood glucose during fasting [44] (Figure 1). There is also a lactate-glucose metabolic cycle between the renal cortex and the medulla: the relatively hypoxic renal medulla mainly relies on glycolysis to produce lactate, and the lactate is transported to the renal cortex for oxidation or gluconeogenesis, after which the renal medulla can reuse the glucose. It is worth noting that during metabolic acidosis, the kidney increases both lactate reabsorption and gluconeogenesis, which helps to clear lactate while also producing bicarbonate to buffer the acidosis. Lactate is converted to glucose, which consumes hydrogen ions (H+) in the body and produces new bicarbonate. This mechanism not only contributes to lactate clearance and energy production but also helps alleviate systemic acidosis [41].

In addition to hepatic and renal metabolism, lactate can also be oxidized and utilized directly as an energy substrate in a variety of tissues that are rich in mitochondria, exhibit high LDH activity and express MCTs. Mitochondrial lactate oxidation has been proposed to involve a mitochondrial lactate oxidation complex (mLOC), which includes monocarboxylate transporter 1 (MCT1), LDH, and components of the mitochondrial respiratory chain [45,46]. Glucose needs to undergo ten steps of glycolysis to produce pyruvate, while lactate only needs one step. Therefore, lactate can serve as a rapid oxidative fuel in tissues with highly active oxidative metabolism, such as cardiomyocytes, skeletal muscle cells and neuronal cells (Figure 1). The myocardium is a representative tissue that directly uses lactate as an energy substrate. Lactate can account for approximately 10–20% of the daily energy consumption of the heart, and the proportion is higher during exercise [36]. In skeletal muscle, different muscle fiber types have metabolic specificity: mitochondria-rich slow-twitch fibers (type I) have strong aerobic oxidation capacity and can directly oxidize lactate, which is a key part of lactate clearance during exercise. In contrast, fast-twitch fibers (type II) are more closely associated with high-intensity anaerobic exercise and are a major source of lactate production during exercise [38]. In the brain, lactate plays an important role in neuronal energy metabolism through the ANLS. Astrocytes take up glucose and undergo aerobic glycolysis to produce lactate, which is then transported to adjacent neurons to support ATP production. Although the brain preferentially uses glucose as its primary fuel, elevated blood lactate levels during strenuous exercise, mild hypoglycemia, or certain cognitive tasks can increase the use of lactate as a supplement or alternative fuel, thereby helping maintain stable cognitive function [14,37,47].

### 2.3. Lactate Shuttle System

The concept of the lactate shuttle integrates lactate production, transport and utilization into a coordinated metabolic framework. It regards lactate as an important and recyclable energy carrier that actively shuttles within and between tissues, linking sites of production with sites of utilization. This concept thus forms a comprehensive metabolic network [36].

Several studies have reported that LDH and MCTs are present on the mitochondrial membrane, supporting the concept of an intracellular lactate shuttle [48]. In the cytoplasm, glycolysis generates pyruvate, which is converted into lactate by cytosolic LDH. Subsequently, lactate is not necessarily exported from cells but is directly transported to the mitochondria via MCTs associated with the mitochondrial membrane. Within mitochondria, lactate can be reconverted to pyruvate by mitochondrial LDH and subsequently enters the TCA cycle, where it undergoes oxidative metabolism to generate ATP. This process is called the intracellular lactate shuttle.

Intercellular lactate transport occurs within tissues, the most well-known example being the ANLS in the brain [49]. This concept challenges the traditional view that neurons rely exclusively on direct glucose utilization and reveals the metabolic division of labor between different cell types in the brain: astrocytes are responsible for ‘fuel processing’, which pre-treats glucose into more readily available lactate and then delivers it to neurons [14,36] (Figure 1). Similarly, oligodendrocytes also support axonal energy demands through MCT1-mediated lactate release, which is taken up by neurons via MCT2, forming an oligodendrocyte-neuron lactate shuttle particularly critical for maintaining the integrity of long motor neuron axons. Within the lactate shuttle framework of intercellular transport, lactate can mediate energy exchange between organs, serving as a carrier between the productive (e.g., working skeletal muscle) and the consuming (e.g., heart or resting muscle fibers) [50]. This is like building an ‘energy internet’, with lactate acting as a circular ‘digital currency’. Its essence is not passive clearance, but the active transport and distribution of energy.

During lactate shuttling, lactate simultaneously transmits key metabolic and environmental signals to surrounding cells through multiple mechanisms, including receptor activation, transcriptional regulation, and epigenetic modification [50]. Emerging evidence suggests that lactate can regulate cell response and coordinate systemic metabolic adaptation under various physiological and pathological conditions such as hypoxia, exercise and tumor development [41].

### 2.4. Lactate Transporters (MCT Family)

MCTs are members of the solute carrier (SLC) family, which are composed of intracellular N- and C-termini and 12 transmembrane helices. These transporters mediate the proton-coupled transport of monocarboxylates such as lactate, pyruvate and ketone bodies across biological membranes. Among the 14 members of the SLC16 family, MCT1 (SLC16A1), MCT2 (SLC16A7), monocarboxylate transporter 3 (MCT3) (SLC16A8), and monocarboxylate transporter 4 (MCT4) (SLC16A3) are capable of proton-coupled transport of monocarboxylates such as L-lactate. Transport mediated by MCTs is bidirectional and is determined by the transmembrane gradients of protons and monocarboxylates across the cell membrane [51,52].

MCT1 is widely expressed in various tissues, and its main function is to mediate the transport of monocarboxylates such as lactate, pyruvate, and acetate across cell membranes [53]. Due to its relatively high affinity for lactate (Km = 3–5 mM), MCT1 can efficiently mediate lactate uptake at physiological concentrations. In oxidative tissues such as myocardium, slow-twitch muscle fibers and neurons, MCT1 facilitates lactate uptake for metabolic utilization [53]. After its production, lactate is transported across cell membranes primarily through MCTs, among which MCT1 plays a major role in oxidative tissues. However, due to the bidirectional transport of MCT, MCT1 can also participate in the efflux depending on local metabolic conditions and transmembrane concentration gradients.

As an important transporter that maintains lactate homeostasis, MCT1 achieves efficient energy recovery by ‘circulating’ lactate from the production site to the consumption site and becomes a bridge connecting different tissue metabolism [54].

The deficiency of MCT1 in skeletal muscle leads to elevated intramuscular lactate levels, reduced glycolytic muscle fibers, and increased glucose flux into the TCA cycle [55]. Evidence also suggests that MCT1 enhances muscle mitochondrial activity by increasing expression of peroxisome proliferator-activated receptor gamma coactivator-1α (PGC-1α), thereby promoting the conversion of muscle fibers to oxidative fiber type, and improves running performance [53,56].

MCT2 is mainly expressed in the nervous system, especially in neurons of the brain and retina [57]. Among the MCT isoforms, MCT2 has the highest affinity for lactate (Km = 0.5–0.8 mM), which means that MCT2 can efficiently take up lactate even at relatively low extracellular concentrations. A key function of MCT2 is to mediate the uptake of lactate into neurons from surrounding glial cells, thereby providing an important energy substrate for neuronal activity [28]. In addition, lactate transported through MCT2 is also involved in the regulation of neuronal excitability and synaptic plasticity, particularly under conditions of high metabolic demand.

The expression of MCT3 is highly restricted and primarily confined to the retinal pigment epithelium (RPE) and choroid plexus epithelium [58]. In these tissues, MCT3 is involved in regulating the transport of lactate across the retinal barrier, helping to maintain metabolic homeostasis and pH balance in the subretinal space [59]. MCT3 functions together with other MCT isoforms, enabling efficient exchange of lactate and related metabolites between tissues during lactate metabolism [58,59].

MCT4 is another key member of the MCT family. MCT4 is highly complementary to MCT1 and primarily mediates lactate efflux from glycolytic cells. Compared with MCT1, MCT4 has a lower affinity for lactate (Km = 22–28 mM), but a stronger transport capacity, which is particularly suitable for exporting lactate from highly glycolytic cells. Its relatively high Km value for pyruvate (approximately 150 mM) ensures that pyruvate is largely retained within the cell. Under these conditions, pyruvate can be reduced to lactate while simultaneously facilitating the oxidation of NADH and regeneration of NAD^+^. If pyruvate were exported from the cell, the conversion process would be impaired, which could ultimately disrupt glycolytic flux [60].

During high-intensity exercise, fast-twitch muscle fibers produce large amounts of lactate through glycolysis. MCT4 promotes the rapid efflux of lactate, thereby preventing excessive intracellular acidification and limiting intracellular pH decline [61]. This helps sustain efficient glycolysis and maintain the ability of muscle fibers to contract continuously. In the CNS, MCT4 is highly expressed in astrocytes and mediates lactate efflux in the ANLS, thereby supporting neuronal energy metabolism. Other cells such as leukocytes and tumor cells usually exhibit extremely high glycolytic rates even under aerobic conditions. In these cells, MCT4-mediated lactate efflux is essential for maintaining glycolytic flux and cellular survival, and inhibition of MCT4 can induce intracellular acidification and suppress tumor growth. Therefore, MCT4 has emerged as an important therapeutic target in cancer treatment [62,63].

### 2.5. Lactate as a Signaling Molecule

Recent studies have demonstrated that lactate is not only an intermediate product of metabolism, but also an important signaling molecule involved in a variety of physiological and pathological processes [36]. As cell surface receptors, lactate receptors can sense extracellular lactate and transmit signals via intracellular signaling pathways. Studies on lactate-mediated signaling have revealed its important roles in regulating intercellular communication, energy metabolism, immune responses, and tissue homeostasis [64].

#### 2.5.1. HCAR1-cAMP Signaling Pathway

Hydroxycarboxylic acid receptor 1 (HCAR1), also known as GPR81, is currently the best-characterized receptor for lactate [65]. As a G protein-coupled receptor (GPCR), HCAR1 is specifically activated by lactate and participates in a variety of metabolic and signaling regulation processes. HCAR1 is considered the primary receptor of lactate and is widely expressed in adipose tissue, endothelial cells, immune cells, and several cell types in the brain. After lactate binds to HCAR1, the receptor couples to Giα/o proteins and leads to inhibition of adenylate cyclase activity, thereby reducing intracellular cyclic adenosine monophosphate (cAMP) levels.

In adipose tissue, activation of HCAR1 inhibits hormone-sensitive lipase activity, thereby suppressing the breakdown of triglycerides into free fatty acids and reducing the release of circulating free fatty acids [66,67]. Lactate acts as a negative feedback regulator through HCAR1 to prevent excessive lipolysis. In the CNS, HCAR1 is involved in the regulation of neurovascular coupling [68]. Activation of HCAR1 can regulate glial cell metabolism, promote neuronal energy homeostasis, and exert neuroprotective effects under certain conditions. Therefore, the lactate–HCAR1 signaling pathway is closely associated with synaptic plasticity and brain functions such as learning and memory [69].

Disruption of lactate-mediated HCAR1 signaling has been associated with increased autism-like behavior [70] and seizure susceptibility [71]. Lactate has also been shown to protect axon and myelin development through HCAR1 signaling in experimental neonatal hypoglycemia [72]. In addition to metabolic regulation, HCAR1 signaling is also involved in the regulation of immune responses [73]. Studies have shown that activation of the lactate-HCAR1 pathway in macrophages and dendritic cells can suppress the expression of pro-inflammatory cytokines such as TNF-α and IL-6, thereby exerting anti-inflammatory and immunomodulatory effects [73,74]. Emerging evidence also suggests that HCAR1 signaling may be involved in tumor progression and immune regulation within the tumor microenvironment [75].

#### 2.5.2. Lactate-HIF-1α Signaling

Lactate has been reported to inhibit the activity of prolyl hydroxylases (PHDs), thereby preventing the ubiquitination and degradation of hypoxia-inducible factor-1α (HIF-1α) and stabilizing HIF-1α. Stabilized HIF-1α subsequently promotes glycolysis, stimulates angiogenesis, and enhances cellular survival, proliferation, and migration [76]. Stabilization of HIF-1α represents one of the key mechanisms by which lactate regulates cellular function. Notably, lactate can induce hypoxia-like cellular responses even under normoxic conditions. During hypoxic adaptation, the HIF-1α pathway participates in a positive feedback loop in which enhanced glycolysis promotes lactate production, while elevated lactate levels further stabilize HIF-1α signaling [77,78]. In addition, HIF-1α can transcriptionally up-regulate MCT4, facilitating lactate export and promoting metabolic adaptation under hypoxic or pathological conditions [79,80,81].

#### 2.5.3. Additional Signaling Pathways Associated with Lactate

In addition to the classical Gi-cAMP signaling pathway, lactate has also been shown to activate other signaling cascades in specific cellular contexts. For example, lactate-HCAR1 signaling has been reported to modulate the PI3K/Akt pathway in certain cell types, thereby affecting oxidative stress by regulating NADPH oxidase activity [36,82].

Another receptor that has been proposed to respond to a lactate-rich acidic environment is GPR132. As a proton-sensing GPCR, GPR132 has been shown to mediate lactate-induced activation of downstream signaling pathways, thereby regulating immune cell migration and inflammatory response, especially within the tumor microenvironment and in chronic inflammation [83]. Lactate accumulation in acidic microenvironments has been suggested to influence GPR132-mediated signaling, which may regulate macrophage polarization and tumor–immune interactions [83,84]. However, whether lactate functions as a direct ligand for GPR132 remains to be fully clarified.

### 2.6. Protein Lactylation

Protein lactylation is a newly identified type of post-translational modification and is considered one of the important mechanisms through which lactate regulates cellular signaling and gene expression [23]. Lactylation was first identified on lysine residues of histone proteins, in which lactate-derived lactyl groups are covalently added to lysine residues, thereby directly linking cellular metabolic status to epigenetic regulation [85,86]. Different from the classical mode in which lactate acts as a “first messenger” by binding to membrane receptors to trigger intracellular second-messenger changes and subsequent phosphorylation cascades [87], the concept of lactylation emphasizes that lactate itself serves as a modifying group that can be directly attached to target proteins through covalent bonds, thereby altering protein structure and function and ultimately regulating gene expression and cellular behavior [88].

At present, most studies are still focused on histone lactylation, while research on non-histone protein lactylation remains at an early stage [89]. Increasing evidence suggests that histone lactylation may be involved in a variety of biological processes, including inflammatory responses, immune regulation, and metabolic reprogramming [23,90,91]. Through these mechanisms, lactylation establishes a potential link between cellular metabolic activity and epigenetic regulation, revealing another pathway by which lactate regulates cellular function. One of the best-characterized examples of lactylation-mediated gene regulation has been observed in macrophages, where elevated intracellular lactate levels promote histone lactylation and subsequently activate the transcription of genes associated with wound healing and anti-inflammatory responses [92,93,94]. Through this mechanism, lactate acts as a metabolic signal that coordinates the transition of macrophages from a pro-inflammatory state to a reparative phenotype. It is worth noting that recent studies have suggested that lactylation may also play an important role in the CNS. When neuronal activity is enhanced or brain lactate levels are elevated, changes in histone lactylation levels can be observed, and these changes may be involved in the regulation of neuronal activity-dependent gene expression [25,95]. In addition, given the important role of neuroinflammation in various neurodegenerative diseases, lactylation-mediated inflammatory regulation may also play potential roles in CNS disorders [96,97].

In terms of molecular mechanisms, studies have suggested that the histone acetyltransferase p300 may be involved in the catalytic process of lactylation, thereby promoting the formation of lactylated histone lysine residues [98,99]. In addition, recent proteomic studies have further revealed that lactylation is not limited to histones but is also widely present on a variety of non-histone proteins, suggesting that lactylation may represent a broader regulatory mechanism linking metabolic status with protein function and cellular signaling [100]. In addition to immune regulation, emerging studies have also shown that lactylation may participate in the regulation of tumor progression, cellular metabolism, and hypoxic adaptation [101].

## 3. Dysregulation of Lactate Metabolism and Lactate-Mediated Signaling in ALS

### 3.1. Altered Lactate Levels and Metabolism in ALS

In ALS, aberrant lactate metabolism has been observed in patient tissues, animal models, and cellular systems [20,102,103,104]. In the early stages of ALS, metabolic networks undergo adaptive remodeling in response to energy stress; however, as the disease progresses, these compensatory mechanisms eventually fail, giving rise to widespread metabolic dysfunction [9,105,106]. Notably, lactate levels vary throughout disease progression in ALS and show considerable heterogeneity across different tissues, disease stages, and experimental models [107,108,109] (Table 1).

#### 3.1.1. Lactate Metabolic Alterations in the Central Nervous System (CNS)

Studies measuring lactate levels in cerebrospinal fluid (CSF), which directly reflects the central microenvironment, have yielded controversial results in ALS patients. Several reports have indicated elevated CSF lactate in ALS patients, supporting the presence of CNS lactate-related metabolic abnormalities [108,110,111]; nevertheless, evidence remains inconclusive, as some studies have found no significant differences compared with controls [112].

Experimental studies have shown dysregulation of lactate-related energy metabolism [106,113]. In asymptomatic SOD1-G93A mice, a significant decrease in brainstem lactate levels was detected, suggesting that lactate metabolic disturbances may occur at a very early stage of disease progression, potentially preceding overt pathological changes such as MN degeneration [107]. As disease advances, region-specific changes in CNS lactate have been reported [114]. In the early disease stage, the activities of key glycolytic enzymes (e.g., hexokinase and phosphofructokinase) and TCA cycle enzymes are significantly increased in spinal MN synaptosomes, likely representing a compensatory response to energy insufficiency. In contrast, LDH activity does not increase proportionally, indicating an imbalance between glucose catabolism and lactate-related metabolism [106]. More critically, in ALS, MCT1 expression is reduced in oligodendrocytes, impairing their capacity to provide metabolic support to axons (including lactate supply), thereby exacerbating the metabolic imbalance between glial cells and MNs. In turn, this metabolic imbalance may further disrupt lactate homeostasis. Thus, lactate dysregulation in ALS not only reflects systemic metabolic remodeling but also indicates impairment of intercellular metabolic support networks [115].

#### 3.1.2. Lactate Metabolic Alterations in the Peripheral Nervous System and Systemic Compartments

Unlike the “elevated” pattern observed in the CNS, resting peripheral blood lactate levels demonstrate a clear prognostic value in ALS. A recent international study has clearly demonstrated that lower resting blood lactate levels predict shorter survival and more pronounced weight loss in ALS patients [104], indicating that resting peripheral lactate may serve as a sensitive biomarker reflecting global nutritional depletion and insufficient metabolic reserve. Consistently, higher plasma lactate levels have been significantly associated with prolonged survival in ALS [111].

When the body transitions from rest to exercise stress, the pattern of lactate behavior reverses. Slowed post-exercise lactate elimination rate—rather than elevated lactate concentration per se—is significantly associated with faster functional decline and disease progression in ALS, serving as a key indicator of peripheral mitochondrial dysfunction and impaired metabolic stress tolerance. However, this effect is primarily confined to the rate of functional decline and does not significantly impact overall survival time [116]. This suggests that low resting lactate reflects systemic metabolic reserve depletion, while impaired post-exercise lactate clearance reveals peripheral tissue failure in coping with metabolic stress—together, these two dimensions delineate the dual disruption characteristics of the systemic metabolic network in ALS.

In line with these human observations, SOD1-G93A mice exhibit profound peripheral lactate metabolic abnormalities, as evidenced by a paradoxical pattern of decreased blood lactate and increased skeletal muscle lactate levels at the terminal stage, along with reduced LDH activity and MCT1 expression. These findings indicate a significant dysfunction of lactate metabolism, highlighting a disruption of the systemic metabolic network and peripheral metabolic reprogramming in ALS [117]. These findings parallel the human observations of low resting lactate and impaired post-exercise lactate clearance, collectively delineating a picture of systemic metabolic network disruption in ALS—characterized in the CNS by glycolysis–TCA uncoupling and glial metabolic support deficits, and in the periphery by metabolic substrate depletion and aberrant stress responses.

#### 3.1.3. Integrated Perspective: Lactate Dysregulation as a Central Hub of Metabolic Reprogramming

**Figure 2:**
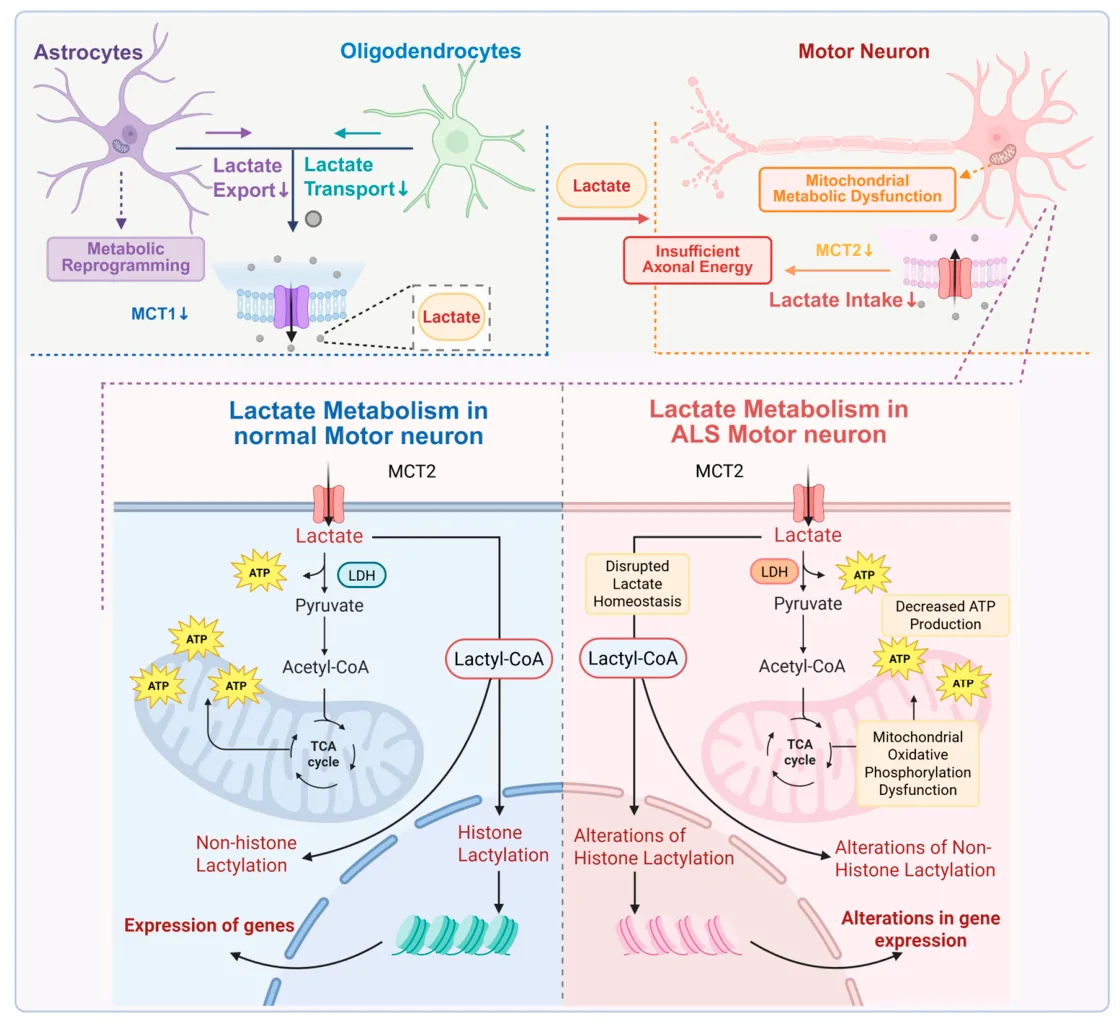
Focusing on lactate shuttling in ALS motor neurons, with a comparison of lactate metabolic patterns between normal and ALS motor neurons. In ALS, a decrease in lactate supply to astrocytes and oligodendrocytes, combined with reduced MCT2-mediated lactate uptake by motor neurons and mitochondrial dysfunction, collectively leads to axonal energy deficiency in the early stages of the disease. Under physiological conditions, motor neurons take up extracellular lactate via MCT2; a small amount of ATP is produced through LDH-catalyzed reactions, while the majority enters the mitochondrial TCA cycle, where oxidative phosphorylation generates large amounts of ATP. Concurrently, lactyl-CoA derived from lactate drives histone and non-histone lactylation modifications, thereby regulating gene expression. In ALS, impaired mitochondrial oxidative phosphorylation leads to a sharp decline in ATP production and an imbalance in lactate homeostasis, which in turn disrupts lactic acid-mediated modifications and triggers abnormal gene expression.

In ALS MNs, lactate dysregulation should not be viewed as an isolated metabolic event, but rather as both a consequence and a potential amplifier of broader metabolic disturbances. Impaired glucose metabolism and altered lipid utilization [118], and mitochondrial dysfunction can disrupt pyruvate oxidation and ATP production, thereby changing the balance between lactate generation, uptake, utilization, and export [119]. In this context, abnormal lactate handling may further disturb cellular redox homeostasis, energy supply, and metabolite-sensitive signaling pathways. In addition, changes in intracellular lactate availability may influence lactate-derived post-translational modifications, such as protein lactylation. However, this possibility remains largely hypothetical in ALS and requires direct validation in MNs and glial cells (Figure 2). Notably, resting and post-exercise blood lactate abnormalities in ALS appear to provide distinct clinical information—one associated with survival, the other with functional decline—but the mechanistic basis for this divergence and whether they reflect independent or shared pathological pathways remain open questions for future investigation.

### 3.2. Disrupted Lactate Shuttle and Lactate Transporter Dysfunction in ALS

Disruption of lactate shuttling between glial cells and neurons is increasingly recognized as an important component of metabolic failure in ALS. Both impaired glial metabolic support and abnormal MCT expression may contribute to defective lactate transfer within the CNS.

Existing studies suggest that abnormalities in ALS astrocytes are not limited to reactive activation and inflammatory phenotypes but also include impaired lactate-mediated metabolic support [120,121]. In mutant SOD1 models, astrocytes have been found to show impaired lactate export-related transport, accompanied by reduced lactate levels in the spinal cord [120] (Figure 2). In vitro experiments further showed that increasing lactate supply could improve MN survival, suggesting that insufficient astrocyte-mediated metabolic support contributes to neuronal injury [120] (Table 1). Studies in TDP-43-related models have further shown that, although aerobic glycolysis and lactate production may be increased in astrocytes, downregulation of MCT1 can restrict lactate export and thereby reduce metabolic support to neurons [121] (Figure 2). Taken together, these findings suggest that disruption of the astrocyte–neuron lactate shuttle in ALS is not simply a matter of “less lactate”, but more likely reflects the coexistence of metabolic reprogramming and impaired lactate release.

**Table 1:** Lactate Metabolism and Transport Disruptions in ALS: Key Findings and Pathological Impacts.

Category	Subcategory	Key Changes in ALS	Evidence Summary	Pathological Implication	Reference
Lactate metabolism	Central nervous system	Elevated CSF lactate reported but controversial; decreased brainstem lactate in presymptomatic mice; glycolysis–TCA uncoupling	Human CSF studies & animal models	Region- and disease stage-dependent dynamic changes in CNS lactate metabolism	[106,107,108,110,111,112,113,114,115]
	Peripheral—resting	Low resting blood lactate predicts shorter survival and weight loss	Human cohort study	Systemic metabolic reserve depletion	[104,111]
	Peripheral—exercise	Slowed post-exercise lactate clearance predicts accelerated functional decline	Human exercise study	Peripheral mitochondrial dysfunction and impaired stress tolerance	[116]
	Peripheral—animal models	Peripheral metabolic reprogramming detectable	animal models	Systemic metabolic network disruption	[117]
Lactate shuttle & transporters	Astrocyte–neuron shuttle	Impaired lactate export despite metabolic reprogramming	In vitro & animal models	Reduced neuronal metabolic support	[120,121]
	Oligodendrocyte support	Decreased MCT1 expression; impaired lactate delivery to axons	Human & SOD1 models	Axonal energy deficit	[115,122,123,124]
	Schwann cell	MCT1 knockout impairs neuromuscular innervation	Animal model	Impaired NMJ innervation maintenance	[125]
	MCT1	Downregulated; early event	Strong evidence	Central node of lactate shuttle dysfunction	[115,122]
	MCT2	Potentially altered	Limited/inconsistent	Possible role in neuronal lactate uptake	[115,126]
	MCT4	Unclear role	Limited evidence	Possible contribution to shuttle dysfunction	[115,126]

By contrast, evidence for impaired oligodendrocyte–axon lactate support in ALS is more direct [115,122]. Oligodendrocytes are a major source of MCT1 and provide axons with lactate and other energy substrates through MCT1-mediated transport. In ALS, MCT1 expression in oligodendrocytes is significantly reduced, which may impair lactate transfer to axons and weaken axonal energy support. This reduction in MCT1 has been observed in both ALS patients and SOD1 mouse models, supporting the view that MCT1 dysfunction may directly impair metabolic coupling between glial cells and neurons. MCT1 downregulation appears to occur early in ALS progression, even before the onset of overt clinical symptoms [115,122] (Figure 2). In addition, oligodendrocyte abnormalities may aggravate MN injury through metabolic and intercellular mechanisms. Mutant SOD1 oligodendrocytes have been shown to induce MN hyperexcitability and death in vitro. Studies further suggest that oligodendrocyte dysfunction is not just a passive consequence of ALS but may actively contribute to disease progression [123]. Although the overall evidence supports the presence of MCT1 dysfunction in ALS, studies using human iPSC-based systems suggest that MCT1-related metabolic defects may vary across experimental models. Some studies have not detected clear changes in lactate release or MCT1 mRNA expression, suggesting that ALS-related pathology may be at least partly model-dependent [124] (Table 1).

Beyond the CNS, the importance of MCT1 in the peripheral nervous system has also been demonstrated. Studies have shown that Schwann cell-specific MCT1 knockout impairs neuromuscular innervation. This suggests that MCT1 plays an important role in maintaining the structural and functional integrity of the neuromuscular junction. Dysfunction of this transporter may therefore exacerbate MN degeneration in ALS by disrupting energy supply to peripheral nerves [125].

MCT2 is primarily expressed in neurons and is an important transporter mediating lactate uptake in the CNS. In some ALS models, abnormalities in neuronal energy metabolism suggest that MCT2-mediated lactate utilization may be affected (Figure 2). However, compared with MCT1, direct evidence for alterations in MCT2 in ALS remains limited and inconsistent, and its precise role therefore still requires further investigation [115,126]. MCT4 is associated with lactate export from glycolytic astrocytes and participates in lactate transport at the neuromuscular junction; it may participate in lactate shuttle dysfunction in ALS, although its specific role remains unclear [125,126] (Table 1).

### 3.3. Dysregulation of Lactate-Related Signaling Pathways in ALS

#### 3.3.1. HIF-1α-Related Signaling Dysregulation in ALS

HIF-1α is a key regulator of cellular responses to hypoxia and metabolic stress. In ALS, the focus is not on the pathway itself, but on disease-related abnormalities in HIF-1α-associated adaptive signaling. Notably, lactate itself can stabilize HIF-1α by inhibiting PHD activity, thereby promoting its nuclear accumulation and subsequent transcription of downstream target genes, constituting a hypoxia-independent pathway of HIF-1α activation. In the context of ALS, this link between lactate accumulation and aberrant HIF-1α signaling is particularly significant, as HIF-1α expression is progressively increased in spinal MNs of ALS model mice. However, despite this accumulation, MNs fail to mount an adequate neuroprotective response through the HIF-1α pathway, which may contribute to neurodegeneration in ALS [127]. In ALS, abnormalities in the HIF-1α pathway appear to extend beyond simple hypoxic stress and reflect a dysregulated adaptive response. Monocytes from patients with sporadic ALS show an abnormal HIF-1 response under hypoxic conditions, suggesting that this protective pathway is already disturbed at the cellular level [128]. In SOD1-G93A ALS model mice, HIF-1α and several downstream targets, including VEGF, HO-1, and EPO, are altered during MN degeneration, indicating that hypoxic adaptation is not properly maintained [127].

Evidence further suggests that HIF-1α dysregulation in ALS involves not only altered expression, but also impaired intracellular transport [129]. In spinal cord tissue from ALS patients, HIF-1α immunoreactivity is increased in the cytoplasm of anterior horn cells, whereas VEGF and its receptors are reduced, indicating a mismatch between HIF-1α accumulation and downstream signaling output [130] (Table 2). A similar pattern is observed in mutant SOD1 mice, where cytoplasmic-to-nuclear transport of HIF-1α is impaired even before overt symptom onset [130].

It is worth noting that these abnormalities are detectable from the early symptomatic stage, suggesting that disruption of HIF-1α-related signaling is involved relatively early in disease progression [127]. In vivo HIF-1α imaging has shown increased hypoxic stress in the spinal cord and lower-limb muscles of ALS mice even at the presymptomatic stage (Table 2). As disease progresses, however, HIF-1α expression declines, suggesting that this adaptive pathway may become progressively impaired over time [129]. Supporting this idea, pharmacological stabilization of HIF-1α with dimethyloxalylglycine (DMOG) reduces hypoxic damage, glial activation, apoptosis, and MN degeneration in ALS mice, while also prolonging survival [129]. In addition to impaired hypoxic adaptation, HIF-1α dysregulation in ALS may also be associated with inflammatory responses in monocyte- and glia-related compartments, further contributing to disease progression [127,128].

#### 3.3.2. BDNF/TrkB-Related Signaling Abnormalities in ALS

In the normal nervous system, exercise can increase circulating mature brain-derived neurotrophic factor (mBDNF), and lactate infusion during exercise can further enhance this response, suggesting that lactate may be involved in exercise-induced BDNF regulation [131]. Previous studies have shown that lactate can enhance NMDAR-mediated signaling and the subsequent increase in intracellular calcium and can also promote Bdnf expression through a SIRT1-dependent pathway involving PGC-1α and FNDC5 [69,132]. Through these effects, lactate-related NMDAR–BDNF signaling may participate in the regulation of synaptic plasticity and neurotrophic support under physiological conditions [69,132].

In ALS, abnormal glutamate metabolism and increased excitotoxicity are common pathological features, and these changes may disturb NMDAR-related signaling [133,134]. At the same time, BDNF/TrkB-related signaling is also altered in ALS [135], although the findings are not fully consistent across different tissues and sample types [136,137]. In spinal cord tissue from ALS patients, the remaining MNs show reduced BDNF-like immunoreactivity together with increased NGF and TrkA signals, suggesting that neurotrophin responsiveness changes during disease progression [136]. However, other studies did not find a clear overall decrease in spinal cord BDNF staining, and clinical studies also suggest that BDNF-related changes may vary between different tissues and compartments [135,137]. Therefore, it may be more appropriate to describe this aspect of ALS as dysregulation of BDNF/TrkB-related signaling, rather than a simple overall decrease in BDNF expression [135,136,137]. Because ALS is also accompanied by a broad metabolic disturbance, mitochondrial dysfunction, and altered lactate metabolism, these changes may further interfere with NMDAR–BDNF-related signaling and thereby impair synaptic plasticity and neuronal repair [133,134] (Table 2). Although the exact role of lactate-related NMDAR–BDNF signaling in ALS still needs further study, its dysregulation may contribute to reduced neurotrophic support and increased vulnerability of MNs during disease progression [133,134,136].

#### 3.3.3. PGC-1α-Related Metabolic Adaptation in ALS

PGC-1α is an important regulator of mitochondrial biogenesis and cellular metabolic adaptation. It helps maintain oxidative metabolism, supports mitochondrial function, and is closely related to tissue responses under metabolic stress. In this sense, the PGC-1α axis is not only linked to energy production, but also to the ability of cells to adjust to changing metabolic demands. Because lactate is increasingly recognized as both a metabolic substrate and a signaling molecule, its interaction with the PGC-1α axis may be relevant to cellular metabolic adaptation [138,139].

In ALS, this adaptive axis appears to be disturbed. In SOD1-G93A mice and in patients with sporadic ALS, PGC-1α and several PGC-1α-regulated factors are altered in the spinal cord and skeletal muscle, with some of the earliest changes detected in muscle, suggesting that impairment of this pathway may occur early and involve both central and peripheral tissues [140]. Experimental studies further show that increasing PGC-1α activity can preserve mitochondrial biogenesis and muscle function, and PGC-1α overexpression has also been reported to exert protective effects on neurons, indicating that disruption of this pathway may contribute to defective metabolic adaptation and neuronal vulnerability in ALS [138,141]. Importantly, lactate has been identified as an inducer of the neuronal PGC-1α system, whereas this CNS-specific response is lost in patient-derived motoneurons carrying FUS mutations [139]. These findings suggest that altered lactate metabolism in ALS may not only reflect metabolic disturbance but may also impair PGC-1α-related adaptive signaling and thereby weaken mitochondrial maintenance and cellular stress resistance [139] (Table 2).

#### 3.3.4. HCAR1-Mediated Lactate Signaling in ALS

HCAR1 is an important receptor involved in the signaling function of lactate in the nervous system. In ALS, however, direct evidence for HCAR1-mediated lactate signaling remains limited [35]. Increasing evidence suggests that ALS and related neurodegenerative conditions are accompanied by altered lactate metabolism [35], while cortical hyperexcitability has been recognized as an early and intrinsic feature of ALS [142,143]. In addition, glutamate transport impairment, excitotoxic stress, and persistent neuroinflammation are also well-established pathological features of the disease [144,145]. These changes provide a biological context in which HCAR1-related signaling may be affected during disease progression. Nevertheless, current studies still do not clearly demonstrate disease-specific changes in HCAR1 expression, localization, or downstream signaling in ALS tissues or models. Therefore, HCAR1-mediated signaling is better described as a potentially relevant but still insufficiently defined component of lactate signaling dysregulation in ALS [35] (Table 2).

#### 3.3.5. Protein Lactylation in ALS

Lactylation represents an important lactate-related mechanism linking cellular metabolism to gene regulation. In ALS, however, direct evidence for abnormal lactylation remains limited [15]. ALS progression is often accompanied by altered lactate metabolism, mitochondrial dysfunction, impaired glucose metabolism, and persistent neuroinflammation, all of which may reshape the metabolic environment required for lactylation-related regulation [146]. Recent reviews of lactate in neurodegenerative diseases have proposed that lactate should be considered not only an energy fuel and signaling molecule, but also a donor for protein lactylation, and ALS has been included in this broader disease framework [35]. In addition, studies in other CNS disease settings suggest that changes in lactylation in immune cells and glial cells are associated with transcriptional regulation of inflammation-related genes, providing indirect mechanistic support for exploring this pathway in ALS [15,96]. Therefore, although direct validation in ALS tissues and models is still lacking, altered lactate homeostasis and metabolic stress may interfere with lactylation-dependent gene regulation and thereby affect neuroinflammatory responses and cellular stress pathways [15]. In this sense, lactylation may represent a potential mechanism linking metabolic disturbance, epigenetic alteration, and neuroinflammation in ALS (Table 2).

## 4. Therapeutic Potential of Targeting Lactate Metabolism in ALS

### 4.1. Metabolic Interventions Influencing Lactate Homeostasis

Increasing evidence suggests that ALS is frequently accompanied by marked systemic energy imbalance. Hypermetabolism, progressive weight loss, and reduced nutritional reserves are common features, and some patients already show increased resting energy expenditure at early disease stages [147,148]. Recent longitudinal evidence has further established a direct link between this systemic catabolic state and aberrant resting lactate metabolism, demonstrating that baseline lactate levels can effectively predict subsequent rates of nutritional decline in patients with ALS [104]. Consequently, although nutritional and metabolic interventions aimed at improving overall energy balance do not directly target lactate production or clearance, they may indirectly reshape lactate homeostasis by optimizing substrate availability and modulating the flux between glycolysis and oxidative phosphorylation [12,147,149,150,151]. Viewed within the framework of lactate metabolism, such interventions in fact constitute important support for delaying metabolic decompensation and provide a clinical context for understanding the dynamic changes in lactate as a prognostic biomarker [104].

**Figure 3:**
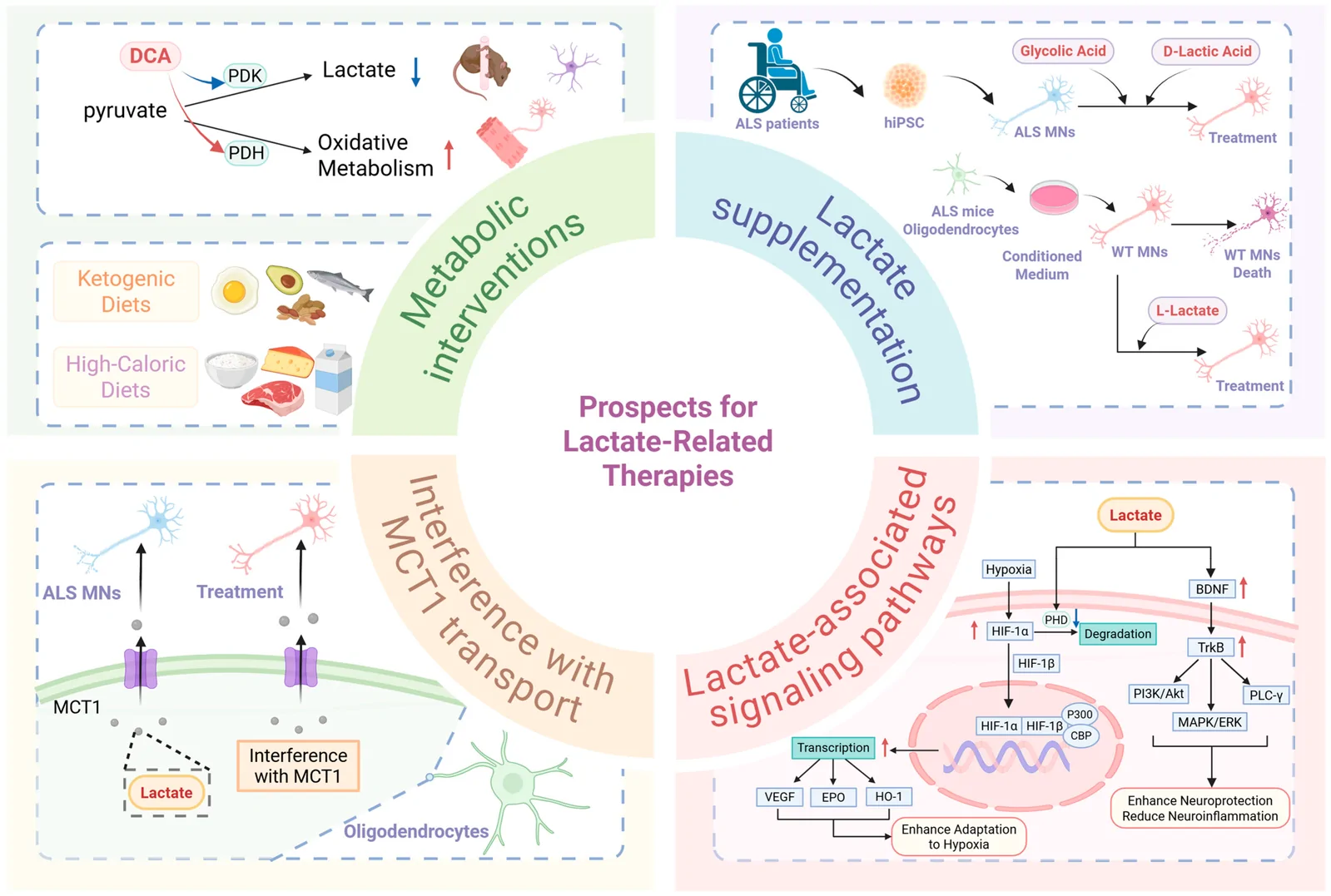
Lactate-related therapies for ALS. DCA exerts a protective effect on motor neurons by rebalancing pyruvate and lactate metabolism and has shown therapeutic potential in ALS models. Both the ketogenic diet and a high-energy diet can increase DCA levels, thereby reducing the lactate metabolic burden on motor neurons. In vitro experiments indicate that glycolic acid and D-lactate can improve the pathological phenotype of motor neurons in ALS patients, while L-lactate also exerts a protective effect on motor neurons in ALS mice. Furthermore, enhancing the efficiency of lactate transport by MCT1 in oligodendrocytes is also considered a potential therapeutic strategy. At the signaling regulation level, lactate activates the HIF-1α pathway by inhibiting PHD, thereby enhancing cellular adaptation to hypoxia; simultaneously, exogenous lactate supplementation can also alleviate neuroinflammation by exerting neuroprotective effects.

Among currently available interventions, dichloroacetate (DCA) exhibits the most direct connection to lactate homeostasis. DCA inhibits pyruvate dehydrogenase kinase (PDK) and activates the pyruvate dehydrogenase complex (PDH), thereby channeling pyruvate into mitochondrial oxidative metabolism and attenuating its conversion to lactate [152]. In the SOD1-G93A model, DCA treatment enhanced mitochondrial coupling in astrocytes, mitigated their toxicity toward MNs, and improved motor function and survival in experimental models [152] (Figure 3). Follow-up studies further demonstrated that DCA ameliorates mitochondrial function in aberrant glial cells, curbs their proliferation and neurotoxicity, and reduces MN degeneration and gliosis in vivo, accompanied by decreased extracellular lactate levels [153] (Figure 3). Collectively, these observations indicate that the neuroprotective action of DCA is primarily attributable to the restoration of oxidative metabolism and the correction of the “Warburg-like” metabolic phenotype characteristic of ALS [154], while the concomitant decline in lactate serves as a marker of this metabolic reprogramming rather than a deleterious event. Hence, the lactate-lowering effect of DCA is not at odds with lactate’s physiological role as an energy substrate; instead, the two represent distinct facets of lactate metabolism operating at pathological and physiological levels.

In addition to DCA, some alternative-fuel strategies can also be included in this group of metabolic interventions. Ketogenic diets promote ketone body production and provide non-glucose energy substrates, which may be especially relevant when glucose utilization is impaired and mitochondrial function is compromised [155]. Preclinical studies in ALS models suggest that ketogenic or related alternative-fuel approaches can improve metabolic status and partially delay motor decline [155] (Figure 3). Besides serving as alternative fuels, ketone bodies may also exert antioxidant and anti-inflammatory effects, which could further reduce cellular stress [155]. Similarly, triheptanoin, which has anaplerotic effects on the TCA cycle, has shown potential to reduce MN loss and delay symptom onset, suggesting that improving TCA cycle function and overall energy supply may help relieve metabolic stress in ALS [156]. These interventions do not directly target lactate itself but may indirectly influence lactate production and utilization by reducing dependence on dysregulated glycolysis and improving oxidative metabolism and substrate use.

By comparison, the link between high-caloric diets and lactate homeostasis is more indirect. Their main role is to compensate for the increased energy expenditure commonly seen in ALS and thereby help maintain systemic energy balance [151] (Figure 3). Available animal and clinical studies suggest benefits for body weight and nutritional status, but any effect on lactate metabolism is more likely to be secondary to improved overall energy supply and reduced metabolic stress [12,151].

Current evidence remains largely preclinical. Therefore, these approaches are better viewed as potential strategies that indirectly modulate lactate homeostasis by reshaping the broader energy metabolic network, rather than as established lactate-targeted therapies [12,155].

### 4.2. Lactate Supplementation as a Therapeutic Intervention

Direct studies on lactate supplementation in ALS are still limited, and the current evidence should therefore be interpreted with caution. Still, the available work suggests that external lactate, or some lactate-related metabolites, may help improve metabolic support under ALS-related conditions [123]. One useful piece of evidence comes from an in vitro study showing that reduced lactate support from ALS oligodendrocytes contributes to MN death. In that study, adding 2 mM lactate increased MN survival, and in some monoculture conditions even produced a complete rescue (Figure 3). This does not mean that lactate supplementation is already an established therapy, but it does suggest that part of the problem may be related to insufficient metabolic support, and that external lactate can partly correct this defect [123].

More recent work has pushed this idea further by looking at lactate-related metabolites rather than lactate alone. A study has reported that glycolic acid and D-lactate improved mitochondrial and lysosomal trafficking defects in patient-derived MNs from both FUS-ALS and SOD1-ALS (Figure 3). In the same study, the combined treatment restored axonal trafficking phenotypes in FUS-ALS MNs, whereas L-lactate was not effective in that setting. These results suggest that, at least in some ALS models, certain lactate-related metabolites may be more effective than standard lactate supplementation itself [31].

There have also been exploratory attempts to use lactate treatment in vivo. In SOD1-G93A mice, lactate alone or together with AMD3100 did not improve survival or disease onset compared with AMD3100 treatment, although some changes in remyelination and astrocyte pathology were observed. These findings suggest that the therapeutic effect of lactate supplementation in vivo may be limited or context-dependent. So at the moment, the evidence does not support a strong therapeutic effect of lactate supplementation by itself, but it does leave open the possibility that metabolic context, delivery strategy, or combination treatment may matter a lot [157].

Current evidence suggests that lactate supplementation in ALS is still an early and developing area. Direct support for L-lactate itself is limited, while D-lactate and glycolic acid currently look more promising in preclinical models. For now, this strategy is better viewed as a potentially useful metabolic intervention rather than an established therapeutic option, and its efficacy and safety in ALS patients still need to be further validated through carefully designed clinical trials [31].

### 4.3. Targeting MCT Family

Among the MCTs, MCT1 currently has the strongest therapeutic relevance in ALS. Most of the available studies have focused on one main question: whether restoring MCT1 can improve metabolic support and slow disease progression. Early evidence showed that loss of oligodendroglial MCT1 is closely linked to axonal and MN vulnerability, which made MCT1 an attractive metabolic target in ALS [115].

This view was further supported by later studies on oligodendrocyte dysfunction. In vitro, ALS-derived oligodendrocytes were shown to induce MN injury together with reduced lactate production and release, suggesting that transporter-related impairment of metabolic support may directly contribute to neuronal damage [123]. These findings further strengthened the idea that restoring MCT1 function may help correct at least part of the metabolic deficit in ALS (Figure 3).

However, when this idea was tested more directly as a treatment strategy, the results were less encouraging. In the most relevant intervention study to date, AAV9-mediated MCT1 gene delivery successfully achieved good oligodendrocyte transduction and MCT1 transgene expression in SOD1-G93A mice; however, this intervention failed to improve disease onset, motor performance, or survival [158]. This negative result is important. It suggests that although MCT1 is a biologically meaningful target, restoring MCT1 expression in oligodendrocytes alone—even when successfully achieved—may be insufficient to counteract the established metabolic and cellular dysfunction in ALS (Figure 3).

There have also been in vivo attempts to intervene along the same metabolic axis. A study reported that upregulation of oligodendroglial MCT1 together with exogenous lactate did not significantly improve disease progression in ALS mouse models, although some changes were observed in remyelination and astrocyte pathology [157]. This again suggests that targeting MCT1 is mechanistically reasonable, but its therapeutic effect may be limited, or at least highly dependent on treatment timing, delivery method, and disease context (Figure 3).

By contrast, the evidence for MCT2 and MCT4 is much weaker. These transporters may also be involved in impaired metabolic support in ALS, but studies directly testing them as therapeutic targets remain very limited. At present, they are better viewed as potentially relevant components of the same metabolic support system, rather than validated therapeutic targets in their own right [122,159].

Current evidence suggests that MCT1 remains the most promising MCT target in ALS, but its therapeutic value is still not established. Available data supports its biological importance but also shows that targeting a single transporter alone may not be enough. Future studies will likely need to place MCT1-based strategies into a broader metabolic treatment framework, rather than expecting a clear therapeutic benefit from restoring transporter expression alone [158].

### 4.4. Lactate-Associated Signaling Pathways as Potential Therapeutic Targets

Lactate may influence ALS progression through several signaling pathways, some of which have shown therapeutic potential. The most substantial evidence currently supports the therapeutic relevance of HIF-1α and BDNF/TrkB signaling, while HCAR1-mediated signaling and protein lactylation remain promising but underexplored in ALS [35,160,161,162,163].

Given the abnormal changes in HIF-1α in ALS, several studies have now attempted to use pharmacological approaches to modulate this pathway to slow the progression of the disease. Among lactate-associated pathways, HIF-1α-related signaling is one of the more practical directions to discuss from a therapeutic point of view. It has been shown that the sustained-release prostacyclin analogue ONO-1301-MS reduced abnormally elevated spinal HIF-1α and its downstream targets EPO and VEGF in SOD1-G93A mice, while also increasing mature BDNF and ATP levels and improving motor function and MN survival at late disease stages [160]. Another study showed that the multitarget iron chelator M30 increased HIF-1α and its target genes, enhanced BDNF expression, delayed disease onset, and prolonged survival in SOD1-G93A mice [164] (Figure 3).

BDNF/TrkB signaling is another pathway with substantial therapeutic evidence in ALS. For example, 7,8-dihydroxyflavone (7,8-DHF), a TrkB agonist, improves motor performance and increases MN survival in SOD1-G93A mice [161]. Furthermore, intramuscular delivery of BDNF-TTC fusion protein delays symptom onset, preserves muscle innervation, and improves spinal MN survival in the same model [165]. Additionally, deletion of the truncated TrkB.T1 receptor in hSOD1-G93A mice significantly delays MN degeneration and postpones muscle weakness [166]. Studies on exercise-based interventions, such as running and swimming, also show improvements in BDNF/TrkB signaling at the neuromuscular junction and reduced MN loss in ALS mice [167] (Figure 3).

In contrast, evidence for HCAR1-mediated signaling as a therapeutic target in ALS remains limited. While HCAR1/GPR81 has been shown to regulate neuronal activity and metabolic feedback, there is currently insufficient direct evidence to confirm that targeting HCAR1 offers therapeutic benefits in ALS [162].

A similar situation applies to protein lactylation. Lactylation has been implicated in linking metabolic states to gene regulation, potentially affecting inflammation, stress responses, and cell fate in ALS [35]. However, studies targeting protein lactylation in ALS are still scarce, making this pathway an emerging area of interest rather than a fully validated therapeutic target.

Taken together, these findings suggest that lactate-related therapeutic strategies in ALS remain promising but are still at an early stage of development.

## 5. Conclusions and Outlook

In summary, growing evidence suggests that lactate is not only a traditional metabolic intermediate but also serves as both an energy substrate and a signaling molecule in the CNS. In ALS, disturbed lactate metabolism, impaired lactate transport and metabolic support between glial cells and neurons, and dysregulation of lactate-associated signaling pathways may contribute to neuronal energy deficiency, increased neuroinflammation, and synaptic dysfunction, thereby increasing MN vulnerability. In recent years, studies on MCTs-mediated lactate transport, HCAR1-dependent signaling, HIF-1α-related metabolic regulation, and protein lactylation have further expanded our understanding of ALS pathogenesis and provided new perspectives for studying this disease from the angle of metabolism and neural energetics.

From a therapeutic point of view, strategies aimed at improving metabolic homeostasis have received increasing attention. These approaches include high-caloric diets, ketogenic diets, exogenous lactate supplementation, and interventions targeting lactate transporters or lactate-associated signaling pathways. Available experimental studies and early exploratory work suggest that these strategies may, to some extent, improve cellular energy metabolism, restore metabolic support, or reduce neuronal injury. However, most of the current evidence still comes from basic and preclinical studies, and robust clinical data remain limited. Therefore, although lactate-related therapeutic strategies show promise in ALS, the field is still at a relatively early stage of development.

Future studies need to further clarify the precise role of lactate metabolism and its related signaling networks in ALS pathogenesis. Particular attention should be given to the stage-specific and cell type-specific effects of lactate, as well as to the interactions among neuron-glia metabolic coupling, neuroinflammatory regulation, and epigenetic changes. At the same time, more rigorous clinical studies are needed to systematically evaluate the safety, efficacy, and therapeutic scope of metabolic interventions targeting lactate pathways in ALS. As our understanding of lactate biology continues to deepen, targeting lactate metabolism and its associated signaling networks may provide new directions for both mechanistic research and metabolic therapeutic development in ALS.

## Figures and Tables

**Table 2:** Alterations in Lactate-Related Pathways and Their Potential Implications in ALS.

Pathway	Physiological Role	Alterations in ALS	Evidence Level	Potential Implication	Reference
HIF-1α	Hypoxia adaptation	Impaired nuclear transport; signaling mismatch	Moderate	Defective stress adaptation	[127,128,129,130]
NMDAR–BDNF	Synaptic plasticity	Dysregulated; context-dependent	Limited-moderate	Reduced neurotrophic support	[69,131,132,133,134,135,136,137]
PGC-1α	Mitochondrial biogenesis	Reduced activity; impaired metabolic adaptation	Moderate	Mitochondrial dysfunction	[138,139,140,141]
HCAR1	Lactate receptor signaling	Not well defined in ALS	Limited	Potential signaling dysregulation	[35,142,143,144,145]
Lactylation	Epigenetic regulation	Not directly validated	Emerging	Link to inflammation and gene regulation	[15,35,96,146]

## Data Availability

No new data were created or analyzed in this study. Data sharing is not applicable to this article.

## Abbreviations

ALS	Amyotrophic lateral sclerosis
MNs	motor neurons
CNS	central nervous system
GLUT3	glucose transporter 3
MCT2	monocarboxylate transporter 2
NAD^+^	nicotinamide adenine dinucleotide
LDH	lactate dehydrogenase
TCA cycle	tricarboxylic acid cycle
MCTs	monocarboxylate transporters
ANLS	astrocyte-neuron lactate shuttle
AD	Alzheimer’s disease
PD	Parkinson’s disease
mLOC	mitochondrial lactate oxidation complex
MCT1	monocarboxylate transporter 1
SLC	solute carrier
MCT3	monocarboxylate transporter 3
MCT4	monocarboxylate transporter 4
PGC-1α	gamma coactivator-1α
RPE	retinal pigment epithelium
HCAR1	Hydroxycarboxylic acid receptor 1
GPCR	G protein-coupled receptor
cAMP	cyclic adenosine monophosphate
PHDs	prolyl hydroxylases
HIF-1α	hypoxia-inducible factor-1α
CSF	cerebrospinal fluid
mBDNF	mature brain-derived neurotrophic factor
DCA	dichloroacetate
PDK	pyruvate dehydrogenase kinase
PDH	pyruvate dehydrogenase complex
7,8-DHF	7,8-dihydroxyflavone
